# Radiomics prognostication model in glioblastoma using diffusion- and perfusion-weighted MRI

**DOI:** 10.1038/s41598-020-61178-w

**Published:** 2020-03-06

**Authors:** Ji Eun Park, Ho Sung Kim, Youngheun Jo, Roh-Eul Yoo, Seung Hong Choi, Soo Jung Nam, Jeong Hoon Kim

**Affiliations:** 1 Department of Radiology and Research Institute of Radiology, University of Ulsan College of Medicine, Asan Medical Center, Seoul, Korea; 2 Department of Radiology, Seoul National University Hospital, Seoul, Korea; 3 Department of Neurosurgery, University of Ulsan College of Medicine, Asan Medical Center, Seoul, Korea; 4 Deparment of Pathology, University of Ulsan College of Medicine, Asan Medical Center, Seoul, 05505 Korea

**Keywords:** Prognostic markers, Outcomes research

## Abstract

We aimed to develop and validate a multiparametric MR radiomics model using conventional, diffusion-, and perfusion-weighted MR imaging for better prognostication in patients with newly diagnosed glioblastoma. A total of 216 patients with newly diagnosed glioblastoma were enrolled from two tertiary medical centers and divided into training (n = 158) and external validation sets (n = 58). Radiomic features were extracted from contrast-enhanced T1-weighted imaging, fluid-attenuated inversion recovery, diffusion-weighted imaging, and dynamic susceptibility contrast imaging. After radiomic feature selection using LASSO regression, an individualized radiomic score was calculated. A multiparametric MR prognostic model was built using the radiomic score and clinical predictors. The results showed that the multiparametric MR prognostic model (radiomics score + clinical predictors) exhibited good discrimination (C-index, 0.74) and performed better than a conventional MR radiomics model (C-index, 0.65, *P* < 0.0001) or clinical predictors (C-index, 0.66; *P* < 0.0001). The multiparametric MR prognostic model also showed robustness in external validation (C-index, 0.70). Our results indicate that the incorporation of diffusion- and perfusion-weighted MR imaging into an MR radiomics model to improve prognostication in glioblastoma patients improved its performance over that achievable using clinical predictors alone.

## Introduction

Glioblastomas are characterized by their morphologic, genetic and gene expression heterogeneity^1^, which leads to resistance to treatment and short term recurrence^2^. Recent immunohistochemistry and genomic sequencing analysis has improved the recognition of that the prognostic biomarkers of isocitrate dehydrogenase (IDH) and O6-methylguanine-methyltransferase (MGMT) promoter methylation are associated with longer survival^3,4^. However, little progress has been made towards non-invasive prediction of survival in patients with glioblastoma.

Radiomics approaches extract high-dimensional features using automated data-mining algorithms^5,6^, and have shown great promise as surrogate measures of the intra-tumoral heterogeneity of genetic features^4^. In particular, radiomics analysis has been successfully applied to prognosis prediction in glioblastomas^7,8^ using conventional MR imaging with T1-weighted imaging (T1WI), T2-weighted imaging (T2WI), fluid attenuation inversion recovery (FLAIR), and contrast enhanced imaging. However, glioblastomas are known to exhibit distinct morphologic characteristics on diffusion-weighted imaging (DWI) or dynamic susceptibility contrast (DSC) imaging, with low apparent diffusion coefficient (ADC) values and high cerebral blood volume (CBV) being related to tumor aggressiveness^9–11^. Furthermore, histogram and texture analyses of ADC or CBV have demonstrated prognostic relevance^12,13^. This opens up the possibility that ADC and CBV maps may provide useful imaging signatures relevant to prognostication using radiomics analysis, signatures that are different to those obtained from conventional MR imaging.

We hypothesized that incorporating ADC and CBV maps into MR radiomics analysis would enhance survival prediction in patients with glioblastoma. We therefore developed a multiparametric MR radiomics model and compared its performance with a conventional MR radiomics model and established clinical variables. Furthermore, we validated its robustness with an external cohort whose data were obtained using different MR acquisition protocols. The purpose of this study was to develop and validate a radiomics model using conventional, DWI, and perfusion-weighted MR imaging for better prognostication in patients with newly diagnosed glioblastomas.

## Methods

### Patients

**Figure 1:**
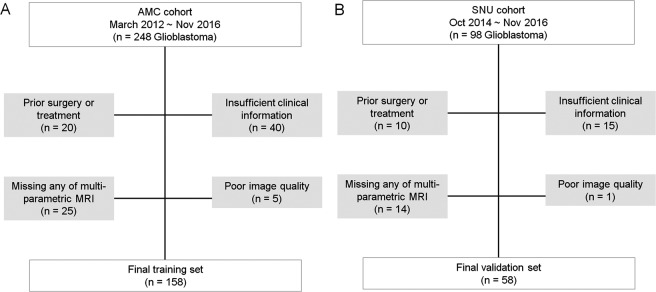
Flow diagram showing the patient selection protocol and the inclusion and exclusion criteria.

The institutional review board of Asan Medical Center and Seoul National University Hospital approved this retrospective study, and the requirement for informed consent was waived. All methods were performed in accordance with the relevant guidelines and regulations. We searched the electronic database of the Department of Radiology at our tertiary hospital and retrospectively reviewed patient records between March 2012 and March 2016. We identified 248 consecutive patients with pathologically confirmed IDH-wild type glioblastoma, according to the 2016 World Health Organization Classification of Tumors of the Central Nervous System^14^. The inclusion and exclusion process is shown in Fig. 1. All patients underwent pretreatment multiparametric MRI including contrast-enhanced T1-WI (CE-T1), FLAIR, DWI, and dynamic susceptibility contrast (DSC) imaging. All patients were treated with concurrent chemoradiation therapy (CCRT). The standard CCRT procedure^2^ consisted of fractionated focal radiotherapy at a dose of 2 Gy per fraction given once daily 5 days per week over a period of 6 weeks, for a total dose of 60 Gy. Concomitant chemotherapy consisted of temozolomide at a dose of 75 mg per square meter per day, given 7 days per week from the first to the last day of radiotherapy, and after a 4-week break patients receive up to six cycles of adjuvant temozolomide according to the standard 5 day schedule every 4 weeks. The dose was 150 mg per square meter and was increased to 200 mg per square meter beginning with the second cycle, providing that there were no toxic effects. The exclusion criteria were as follows: patients had a prior history of surgical treatment (n = 20), had insufficient clinical information (n = 40), or any of multi-parametric imaging data was missing (n = 25) or unreadable (because of an artifact) (n = 5). These steps yielded 158 consecutive patients (mean age, 59.5 years old; male - female ratio, 96:62). This cohort was used as a training set to develop a radiomics model for prognostication in patients with glioblastoma. Identical inclusion criteria were used to SNU cohort obtained between October 2014 and November 2016. From a total of 98 patients, patients were excluded if they had a prior history of surgical treatment (n = 10), had insufficient clinical information or did not receive CCRT (n = 15), or if any of the multiparametric imaging data was missing (n = 14) or of poor image quality (n = 1). Total 58 patients with glioblastoma were finally included and used for external validation of the model.

### Imaging data acquisition and post-processing

**Figure 2:**
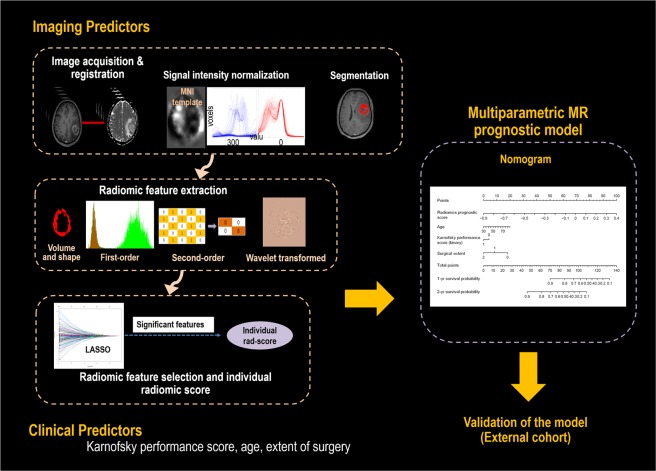
Analysis pipeline for this study. The imaging analysis includes acquisition, co-registration, signal intensity normalization for conventional magnetic resonance imaging data, and segmentation. A Cox regression with least absolute shrinkage and selection operator method (LASSO) was applied to select significant radiomic features. The individualized radiomic score is calculated as the sum of each radiomic variable multiplied by a non-zero coefficient from LASSO. Subsequently, a composite prognostic model was built using the features showing a significant association, including the individual radiomic score and clinical predictors.

The accrual process used for developing the model is summarized in Fig. 2. In the training set, all MRI studies were performed on the same 3-T unit (Achieva; Philips Medical Systems, Best, The Netherlands) using an eight-channel head coil. The brain-tumor imaging protocol of our institution includes the following sequences: T2WI, FLAIR imaging, T1WI, DWI, CE-T1, and DSC perfusion MRI. DWI was acquired in three orthogonal directions, and the images were combined into a trace image. DWI was obtained using the following parameters: repetition time (TR)/echo time (TE), 3000/56 ms; diffusion gradient encoding, b = 0, 1000 s/mm^2^; field of view (FOV), 25 cm; slice thickness/gap, 5 mm/2 mm; matrix, 256 × 256; and acquisition time, 39 s. A contrast-enhanced high-resolution anatomical three-dimensional (3D) volume image was obtained using a gradient-echo T1-weighted sequence with the following parameters: TR/TE, 9.8/4.6 ms; flip angle, 10°; FOV, 256 mm; matrix, 512 × 512; and slice thickness, 1 mm with no gap. DSC perfusion MRI was performed using a gradient-echo, echo-planar sequence during the administration of a standard dose of 0.1 mmol/kg gadoterate meglumine (Dotarem; Guerbet, Paris, France) at a rate of 4 mL/s using a MRI-compatible power injector (Spectris; Medrad, Pittsburgh, PA, USA). The bolus of contrast material was followed by a 20 mL bolus of saline administered at the same injection rate. The parameters for DSC MRI were as follows: TR/TE, 1808/40 msec; flip angle, 35°; FOV, 24 cm; slice thickness/gap, 5 mm/2 mm; and matrix, 128 × 128. The total acquisition time for DSC MRI was 1 min 54 s. We applied double dose protocol, where a preload of 0.1 mmol/kg gadoterate meglumine was given before the dynamic bolus, and then the dynamic bolus was administered as another 0.1 mmol/kg gadoterate meglumine (Dotarem; Guerbet) delivered at a rate of 4 mL/s by an MRI-compatible power injector (Spectris; Medrad). The bolus of contrast material was followed by a 20 mL bolus of saline, injected at the same rate. The full preload bolus was intended to optimize protocol for CBV estimation^15^.

The brain tumor MRI in the validation set was also acquired on a 3-T scanner, and included T2WI, FLAIR, T1WI, DWI, CE-T1, and DSC perfusion imaging sequences. The acquisition protocols used in the study are shown in Supplementary Information 1.

### Imaging post-processing

The apparent diffusion coefficient maps were calculated using the b values of 0 and 1000 s/mm^2^, using a two-point estimate of signal decay: ADC = −ln (*S*[*b*]*/S*[0])*/*b, where b indicates the b value and S(0) and S(b) are the signal intensities of images with b values at 0 and 1000, respectively. The post-processing of the DSC imaging was performed using commercial software (NordicICE; NordicNeuroLab, Bergen, Norway). Leakage correction was performed using the method of Weisskoff *et al*.^16^ with further adaptations from Boxerman *et al*.^17^, with leakage being estimated from the deviation in each voxel according to a non-leakage reference tissue response curve. After correction for contrast agent leakage, the whole-brain relative CBV was calculated using a numerical integration of the time concentration curve. Next, we normalized the relative CBV (nCBV) images with the mean intensity of the contralateral normal-appearing cerebral white matter at the centrum semiovale, which was manually selected by a researcher (Y.H.J., with 2 years’ of experience in neuroimaging processing). The diameter of the selected region of interest (ROI) was 4 mm. The nCBV maps were created by dividing each CBV value by the contralateral ROI on a pixel-by-pixel basis.

For 3D CE-T1WI and FLAIR data, signal intensity normalization was used to remove noise and reduce variance in the T1-based signal intensity of the brain. We applied the hybrid white-stripe method^18^ for intensity normalization using the ANTsR and WhiteStripe packages^19,20^ in the R software package (R Foundation for Statistical Computing, Vienna, Austria, URL: http://www.R-project.org, 2016). This incorporates processes of the statistical principles of image normalization, preserving ranks among tissue and matching the intensity of tissues without upsetting the natural balance of the tissue intensities^20^. Before feature extraction, we excluded outlying image intensities from the ADC and nCBV maps by excluding those voxels inside the ROI with a value outside ± 3 standard deviations of the mean^21^.

The ADC and nCBV maps were then co-registered to the 3D contrast-enhanced T1WI using SPM software (www.fil.ion.ucl.ac.uk/spm/). The co-registration process included the generation of a brain mask from 3-dimensional (3D) CE-T1WI and transformation of the ADC and nCBV maps of each patient to the brain-extracted 3D CE-T1WI volume using affine transformations with normalized mutual information as a cost function^22^, with 12 degrees of freedom and tri-linear interpolation.

Segmentation of the enhancing tumor region was performed by a neuroradiologist (with 4 years of experience in neuro-oncological imaging) who semi-automatically defined the region on the 3D CE-T1 using a segmentation threshold and region-growing segmentation algorithm implemented using MITK software (www.mitk.org German Cancer Research Center, Heidelberg, Germany)^18^. All segmented images were validated by an experienced neuroradiologist (with 18 years of experience in neuro-oncologic imaging). Finally, we resampled all images into a uniform voxel size of 1 × 1 × 1 mm.

### Radiomic feature extraction

Radiomic features were extracted using Matlab R2014b (The Mathworks, Natick, MA), in accordance with previous studies^8,23^. Details of the radiomic feature extraction are provided in the Supplementary Information. Briefly, the radiomic features consisted of four feature groups: seven volume and shape features, 17 first-order features, 162 texture features, and 1432 wavelet features. The volume and shape features were obtained from the segmented mask and the first-order, texture, and wavelet features were estimated using signal intensity. Then first-order features and texture features were calculated from the eight wavelet decomposition images, which resulted in 1432 wavelet features ([17 + 162] × 8). In total, 1618 features (17 first-order statistics, seven volume and shape-based features, 162 texture features, and 1432 wavelet features) were obtained. For each patient, 1618 radiomic features were extracted from the T1CE, FLAIR, ADC, and CBV data, resulting in a total of 6472 extracted features. Finally, all radiomic features were *z* transformed for group comparisons. The processing time for extraction of the 6472 features was approximately 8 min per patient.

### Radiomic feature selection

We used ${L}_{1}$-penalized estimation for a Cox regression and the least absolute shrinkage and selection operator (LASSO) method^24,25^ to select the radiomic features for prognostication. Briefly, the LASSO is a data analysis method that selects features by fitting a Cox regression model via penalized maximum likelihood estimation. An individualized radiomics score was developed using the non-zero coefficients of the radiomic features. The score is calculated as the sum of each radiomic feature multiplied by a non-zero coefficient from LASSO. The R software and “glmnet” package were used for the LASSO Cox regression model analysis.

### Individualized radiomics score

An individualized radiomics score was developed using the non-zero coefficients of the radiomic features. This score is calculated as the sum of each radiomic feature multiplied by a non-zero coefficient from LASSO according to the equation below.

$$\begin{array}{rcl}{\rm{Radiomics}}\,{\rm{score}} & = & {\rm{coefficient}}\,{\rm{of}}\,{\rm{the}}\,{1}^{{\rm{st}}}\,{\rm{feature}}\times {\rm{value}}\,{\rm{of}}\,{\rm{the}}\,{1}^{{\rm{st}}}\,{\rm{feature}}\\  &  & +{\rm{coefficient}}\,{\rm{of}}\,{\rm{the}}\,{2}^{{\rm{nd}}}\,{\rm{feature}}\times {\rm{value}}\,{\rm{of}}\,{\rm{the}}\,{2}^{{\rm{nd}}}\,{\rm{feature}}\\  &  & +\,{\rm{coefficient}}\,{\rm{of}}\,{\rm{the}}\,{3}^{{\rm{rd}}}\,{\rm{feature}}\times {\rm{value}}\,{\rm{of}}\,{\rm{the}}\,{3}^{{\rm{rd}}}\,{\rm{feature}}\\  &  & +\ldots +\,{\rm{coefficient}}\,{\rm{of}}\,{\rm{the}}\,{{\rm{n}}}^{{\rm{th}}}\,{\rm{feature}}\times {\rm{value}}\,{\rm{of}}\,{\rm{the}}\,{{\rm{n}}}^{{\rm{th}}}\,{\rm{feature}}\end{array}$$

The radiomics score was calculated separately using both conventional radiomic and multiparametric radiomic features obtained from conventional MR imaging, ADC maps, and CBV maps. Next, the radiomics score was used as an imaging predictor for the prognostication model.

### Clinical predictors and outcome definition

Preoperative clinical predictors including the baseline characteristics of sex, age, Karnofsky performance score (KPS; binary, score ≥ 80 or <80), tumor location, tumor volume, MGMT promoter status, and extent of surgery (biopsy, partial resection, or gross total resection) were collected.

The primary endpoint of the study was overall survival (OS), which was calculated from the day of histopathologic diagnosis until the day of death, as obtained from the national health care data linked to our hospital. Patients who were alive at the time of analysis (n = 36, 22.8% in the training set and n = 8, 13.9% in the validation set) were right-censored data and included in the analysis. All patients were followed up every 3–6 months after surgical treatment. The minimum follow-up time to ascertain survival was 1.8 year.

### Statistical analysis

Frequencies and proportions are reported for categorical variables, and the mean and standard deviation for continuous variables. Differences between categorical variables and differences between continuous variables were assessed using the chi-square test and independent *t*-test, respectively.

The model development and validation methods in our study adhered to the Transparent Reporting of a multivariable prediction model for Individual Prognosis or Diagnosis (TRIPOD) statement^26^. For clinical predictors, univariate Cox proportional hazard regression analysis was used to test the association between OS and clinical predictors including sex, age at diagnosis, KPS, tumor location, and tumor volume.

For the radiomics-based risk assessment, a linear predictor of the radiomics score was calculated as a weighted sum of the covariates in the Cox proportional hazard model, where the weights were the regression coefficients^27^. The optimal cutoff for the radiomics score to stratify high- and low-risk groups was estimated using maxstat in R. Prognostic performance was calculated using 10-fold cross validation, which ensured unbiased prediction within the sample^28^. Kaplan-Meier survival curves were constructed, and the significance for stratifying low- and high-risk groups was calculated with a log-rank test. The performance of the prognostic model was quantified with respect to discrimination and calibration^29^. Discrimination was measured with Harrell’s concordance probability index (C-index). The “compareC” package in R was used to compare the C-index values across different models. Calibration was tested using the D’Agostino-Nam version of the Hosmer-Lemeshow test^30^.

A nomogram was built to visualize a multiparametric MR prognostication model using radiomics score and clinical predictors. Statistical analyses were performed using R statistical software (R version 3.3.3, R Core Team, Vienna, Austria). A *P* value less than 0.05 was considered statistically significant.

## Results

The clinical characteristics of the training and validation cohorts are summarized in Table 1. No differences in sex, age, treatment regimen, tumor location, initial KPS, and MGMT promoter methylation status, were found between the training and validation cohorts. The median follow-up times were 2.86 years in the training set and 4.47 years in the validation set. The median survival was 646 days in the training set and 700 days in the validation set.

**Table 1:** Clinical characteristics of the study patients.

Parameter	Training set (n = 158)	External validation set (n = 58)	*P value*
Sex, n			0.52
Male/Female	96/62	38/20	
Age, years			0.27
Median (range)	59.5 (31–83)	57.6 (20–80)	
**Primary treatment, n [%]**			
Extent of resection			0.12
Gross-total resection	72 (45.6%)	34 (58.6%)	
Subtotal resection	57 (36.1%)	19 (32.8%)	
Biopsy	29 (18.4%)	5 (8.6%)	
**Adjuvant treatment**			
RT + TMZ	141 (89.2%)	58 (100%)	0.07
**Other**			
RT only	1 (0.6%)	0	
TMZ only	4 (2.5%)	0	
No RT or TMZ	12 (7.6%)	0	
Location			0.62
Frontal or temporal	73 (46.2%)	29 (50%)	
Others	85 (53.8%)	29 (50%)	
KPS at treatment initiation, n (%)			0.64
≥70	138 (87.3%)	52 (89.7%)	
<70	20 (12.6%)	6 (10.3%)	
**MGMT promoter status, n (%)**			
Methylated	12 (7.6%)	28 (48.3%)	0.13
Unmethylated	25 (15.8%)	19 (32.7%)	
NA	120 (75.9%)	11 (19.0%)	NA
Median follow-up time, years range)	2.86 (1.06–5.67)	4.47 (3.44–6.18)	0.047

Abbreviation: KPS, Karnofsky performance score; CCRT, concurrent chemoradiation therapy; RT, radiation therapy; TMZ, temozolomide; MGMT, O6-methylguanine-DNA-methyltransferase gene methylation status;NA, information not available.

### Significant clinical predictors

Among the clinical predictors, older age (hazard ratio 1.02, *P* = 0.039) and lower KPS at treatment initiation (hazard ratio 1.70, *P* = 0.043) were significant clinical predictors of shorter survival, while gross total resection rather than biopsy/partial resection was associated with longer survival (hazard ratio 0.67, *P* = 0.0004; Supplementary Table 2). The performance of the clinical predictors including age, KPS, and extent of surgery had a C-index of 0.67 (95% CI, 0.64–0.70) in the training set and 0.63 (95% CI, 0.59–0.65) in the validation set.

### Significant radiomic features to calculate a radiomics score

Six significant multiparametric MRI radiomic features were selected using LASSO penalization applied to the training set (Supplementary Fig. 1). The individualized radiomics score was calculated using the corresponding coefficients of each feature according to the equation described below. Three of thesefeatures are from conventional MRI (1 T1CE and 2 FLAIR), one from ADC, and two from CBV maps (Table 2).

$$\begin{array}{rcl}{\rm{Radiomics}}\,{\rm{score}} & = & -\,0.07896580\times [{\rm{T}}1{\rm{CE}}\_{\rm{Sum}}\,{\rm{entropy}}\,({\rm{mean}})\,{\rm{LLH}}\,{\rm{GLCM}}\,{\rm{dist}}=3]\\  &  & -\,0.06340327\times [{\rm{FLAIR}}\_{\rm{Mean}}\,{\rm{absolute}}\,{\rm{deviation}}\,{\rm{LHH}}\,{\rm{first}}\,{\rm{order}}]\\  &  & -\,0.09125977\times [{\rm{FLAIR}}\_{\rm{High}}\,{\rm{gray}}-{\rm{level}}\,{\rm{run}}\,{\rm{emphasis}}\,({\rm{std}})]\\  &  & -\,0.05745977\times [{\rm{ADC}}\_{\rm{SkewnessHHHfirstorder}}]\\  &  & +\,0.03145506\times [{\rm{CBV}}\_{\rm{Entropy}}\,({\rm{std}})\,{\rm{HHL}}\,{\rm{GLCM}}\,{\rm{dist}}\,=\,1]\\  &  & -\,0.08185888\times [{\rm{CBV}}\_{\rm{Long}}\,{\rm{run}}\,{\rm{high}}\,{\rm{gray}}-{\rm{level}}\,{\rm{emphasis}}\,({\rm{mean}})\,{\rm{HHH}}\,{\rm{GLRLM}}]\end{array}$$

**Table 2:** Selected radiomic features in the multiparametric MRI imaging and in each MRI.

Result category	CET1	FLAIR	ADC	CBV
Individual features	Sum entropy (mean) LLH GLCM dist = 3 (*P* = 0.0003)	Mean absolute deviation LHH first order (*P* = 0.0023) High gray-level run emphasis (std)(*P* = 0.0003)	Skewness HHH first order (*P* = 0.0008)	Entropy (std) HHL GLCM dist = 1 (*P* = 0.0014) Long run high gray-level emphasis (mean) HHH GLRLM (*P* = 0.0003)

P-value for each radiomic feature associated with outcome was calculated using univariate Cox proportional hazards regression.

Abbreviations: CET1 = contrast-enhanced T1-weighted imaging, FLAIR = fluid-attenuated inversion recovery, ADC = apparent diffusion coefficient, CBV = cerebral blood volume. H = high-pass filter, L = low-pass filter, GLCM = gray-level co-occurrence matrix, GLRLM = gray-level run-length matrix.

**Figure 3:**
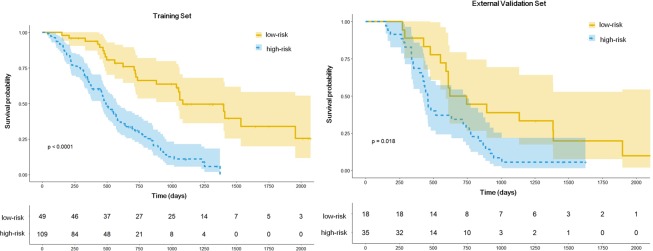
Kaplan-Meier survival curves in the training (**A**) and validation (**B**) sets stratified based on the radiomic prognostic score. Survival curves demonstrate patients with low- and high-risk computing radiomic prognostic score.

The optimal cutoff for stratifying the low- and high-risk groups was −0.07. Figure 3 demonstrates the performance of the radiomic score using the optimal cutoff for survival prediction in both training and external validation sets. The log-rank tests for the Kaplan Meier survival curves were significant for both training (*P* < 0.0001) and external validation sets (*P* = 0.018).

The C-indexes for the radiomics score were 0.69 (95% CI, 0.66–0.72) in the training set and 0.64 (95% CI, 0.59–0.68) in the validation set. The performance of the individual radiomics score using conventional MRI had a C-index of 0.65 (95% CI, 0.60–0.69), which was lower than that of the multiparametric MRI.

### Performance of the multiparametric MR prognostic model and visualization with a nomogram

**Figure 4:**
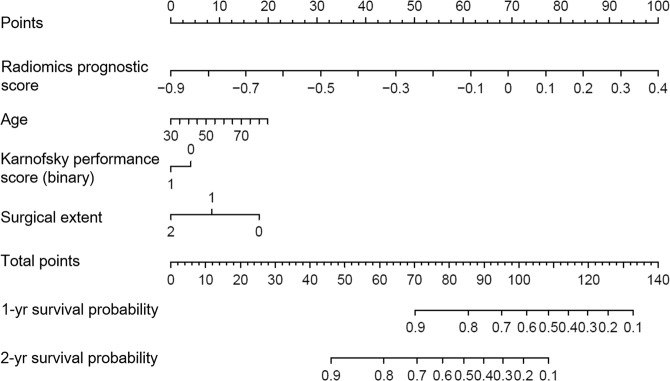
A nomogram predicting the probability of 1 and 2-year survival in patients with glioblastoma. Nomogram includes baseline features including radiomics score, age, and Karnofsky performance score and extent of surgery.

We constructed a composite prognostic model using the radiomic prognostication score and the significant clinical predictors. Visualization of this model with a nomogram is shown in Fig. 4. The diagnostic performance of MR prognostic models is shown in Table 3. The performance of the composite prognostic model (radiomics score + clinical predictors) in the training set had a C-index of 0.74 (95% CI, 0.71–0.77) and showed good calibration (Hosmer-Lemeshow test, *P* > 0.05). This performance was significantly better that that of the radiomics score alone (C-index, 0.69 [95% CI, 0.66–0.72]; *P* = 0.004) and the to clinical predictors (C-index, 0.66 [95% CI, 0.63–0.69]; *P* = 0.004).

**Table 3:** Comparison of prognostic models combining multi-parametric radiomics features for predicting overall survival in the training and the validation set.

	Refined Model	Single Model		
	Multiparametric MR radiomics score + clinical predictors	Multiparametric MR radiomics score	Conventional MR radiomics score	Clinical predictors
**Training set**				
C-index	**0.74**	0.69	0.65	0.67
	Difference	0.057	0.09	0.075
	*P-value*	**0.004**	**<0.0001**	**0.004**
**Validation set**				
C-index	**0.70**	0.64	0.56	0.63

*Note*: baseline clinical predictors are age, Karnofsky performance score, and extent of surgery. *p*-value refers to the significance in the difference of C indices between the combined model and the single model using “CompareC” in R statistical package.

The trend of good discrimination shown by the prognostic model remained when in was applied to the external validation set, with a C-index of 0.70 (95% CI, 0.65–0.72). The model was better able to predict OS than the radiomics score or clinical predictors, although the differences were not statistically significant. The model was well calibrated in the external validation (Hosmer-Lemeshow test, *P* > 0.05).

## Discussion

In this study, we developed and validated a multiparametric MR radiomics model to predict survival in patients with newly diagnosed glioblastoma. The model incorporates six features from the radiomic score and important clinical predictors including age, KPS, and extent of surgery. The multiparametric MR radiomic features provided better performance than conventional MR imaging or single imaging involving ADC or CBV maps, and showed improved prognostic value over a well-established clinical model. Moreover, visualization with a nomogram provides an easy-to-use prognostication model and facilitates personalized outcome prediction in patients with newly diagnosed glioblastoma.

Our model was designed to use an individualized radiomic score to improve prognostication in patients with glioblastoma who will be treated with standard CCRT therapy. Despite studies demonstrating that MR imaging data can predict survival in patients with glioblastoma^31,32^, the use of MR imaging to determine the prognosis in the clinic is still very limited. In this study, the radiomic score improved survival prediction over clinical predictors, thereby demonstrating the potential of imaging phenotypes for predicting survivalin patients with newly diagnosed glioblastoma. Calculation of the radiomics score provides a tool to estimate survival probability at 1 and 2-years, and may help to guide patients who undergo surgery followed by standard CCRT treatment and adjuvant TMZ therapy^2^.

The six multiparametric MR radiomic features selected in this study clearly disciminated outcomes in the both the training set (C-index, 0.691) and validation set (C-index, 0.617). Furthermore, the application of multiparametric MR radiomic features resulted in the higher performance than achieved with conventional MR imaging. Previous prognostic studies using radiomics analysis relied on conventional MRI, and included spatial relationships and voxel intensity information, but did not use ADC and CBV maps, which can reflect the physiological linkage to imaging phenotypes. ADC represents tumor cellularity and CBV represents tumor vascularity, and skewness of ADC^33^ and texture analysis of CBV^13^ have shown prognostic value in newly diagnosed glioblastoma. However, there are concerns that MR-based radiomic features may be vulnerable to changes in acquisition parameters, as the tumor margin and signal-to-noise ratio can easily vary across conventional MRI imaging protocols according to different intensity normalization methods^34^. Also, different acquisition and processing methods can result in differences on CBV maps^35^. This may explain the lower performance on the external validation set compared with the training set. ADC maps may be more robust across the different acquisition^36^ schemes, but this issue needs to be further studied.

To date, radiomic studies have rarely been validated on an external cohort, and the generalizability of the studies has been limited. Our model is strengthened by its validation with an external cohort having heterogeneous MR acquisition protocols and scanner manufacturers. Furthermore, the survival prediction using the radiomic score extends to the individual patient, which fits with the current trend of personalized medicine. The nomogram method could also act as a decision-making support tool before treatment using the variables age, KPS, radiomic prognostication score, and extent of surgery. Our results are in accord with a recent study utilizing a nomogram for glioma grading^37^, which showed that incorporation of a nomogram-derived prediction is useful for radiomics analysis.

This study is subject to a number of limitations. First, only a small number of patients were included, especially in the validation set, which results from the acquisition of DWI and DSC imaging in a single session still not being widely available. Second, important molecular changes such as MGMT promoter methylation status were not considered in this analysis, and MGMT promoter methylation status is a strong predictor of benefit from TMZ therapy^38,39^. Third, although we tested the influence of MR radiomics using different scanning parameters at 3.0 T, testing with a 1.5 T system must be completed before our radiomics approach can be used as a multicenter imaging biomarker with scanners of both strengths. Finally, the radiomics approach consists of data-driven analysis, and thus the biological meaning of the radiomics data is often unclear. This can become an obstacle in clinical practice, along with the necessary labor-intensive image processing and data analysis procedures involved. Although the averaged feature extraction time required for each patient was 8 minutes, further efforts to reduce the time and simplify the analysis will be valuable if the method is to be incorporated into clinical routine.

In conclusion, the multiparametric MR radiomics nomogram improved prognostication for patients with newly diagnosed glioblastoma in comparison with an existing clinical model, both before and after standard treatment regimens. This nomogram was validated externally and showed robustness. To confirm its value in individual prognostication, further prospective trials performing per-patient predictions and incorporating molecular and genomic changes, are required.

## Supplementary information

Supplementary Data.

## Data Availability

The datasets generated during and/or analyzed during the current study are available from the corresponding author on reasonable request.
