# Supplementary material for: Radiomics prognostication model in glioblastoma using diffusion- and perfusion-weighted MRI
Source: Sci Rep. 2020 Mar 6;10:4250. doi: 10.1038/s41598-020-61178-w (PMC7060336; doi:10.1038/s41598-020-61178-w)
Supplement: Supplementary file 1 — Supplementary Data. [file 41598_2020_61178_MOESM1_ESM.docx]

Supplementary data for

**Radiomics prognostication model in glioblastoma using diffusion- and perfusion-weighted MRI**

Ji Eun Park^1^, Ho Sung Kim^1^, Youngheun Jo^1^, Roh-Eul Yoo^2^, Seung Hong Choi^2^, Soo Jeong Nam^3^, Jeong Hoon Kim^4^

^1^Department of Radiology and Research Institute of Radiology, University of Ulsan College of Medicine, Asan Medical Center, Seoul, Korea

^2^Department of Radiology, Seoul National University Hospital, Seoul, Korea

^3^Department of Neurosurgery, University of Ulsan College of Medicine, Asan Medical Center, Seoul, Korea

^4^Deparment of Pathology, University of Ulsan College of Medicine, Asan Medical Center, Seoul 05505, Korea

Correspondence to [radhskim@gmail.com](mailto:radhskim@gmail.com)

1. **Supplementary 1:** Imaging acquisition protocols and **supplementary table 1** for comparison of MR imaging parameters of patients in training set and in external validation set of another tertiary center using the 3-T system
2. **Supplementary information: extraction of radiomic features**
3. **Supplementary Table 2.** Selection of clinical predictors from the training set using a univariate Cox hazard regression model.
4. **Supplementary Figure 1.** Multiparametric magnetic resonance radiomics feature selection using the least absolute shrinkage and selection operator (LASSO) Cox regression model.

.

**Supplementary 1.**

**Supplementary Table 1.** Comparison of MR imaging parameters of patients in training set and in external validation set of another tertiary center using the 3-T system.

| Pulse Sequence | Training cohort | External validation cohort |
| --- | --- | --- |
| Post-contrast T1 |  |  |
| Repetition Time (ms) | 9.8 ± 0.2 (9.0–10.1) | 9.6 ± 1 (8.6–10.4) |
| Echo Time (ms) | 4.6 ± 0.2 (4.4–4.8) | 4.2 ± 0.7 (3.5–4.7) |
| Pixel size (mm) | 0.5 | 0.45 ± 0.02 (0.43–0.47) |
| Section thickness (mm) | 0.5 | 1 |
| FLAIR |  |  |
| Repetition Time (ms) | 9250 ± 433 (9000-10000) | 8962.9 ± 77.3 (8802-9000) |
| Echo Time (ms) | 128.8 ± 4.8 (125–135) | 102.1 ± 10.5 (97-124) |
| Pixel size (mm) | 0.76 ± 0.14 (0.65–0.94) | 0.68 ± 0.2 (0.55–0.89) |
| Section thickness (mm) | 4.5 ± 0.3 (4–5) | 5 |
| DWI |  |  |
| Repetition Time (ms) | 3750 ± 433 (3000–4000) | 8430 (6900–12,000) |
| Echo Time (ms) | 59.4 ± 2.1 (56–61.7) | 66.8 ± 14.2 (55–81) |
| Pixel size (mm) | 1.9 ± 0.04 (1.88–1.97) | 0.83 ± 0.11 (0.75–0.94) |
| Section thickness (mm) | 4.4 ± 0.5 (4–5) | 3.3 ± 1.3 (3–5) |
| DSC |  |  |
| Repetition Time (ms) | 1781 ± 32.6 (1726–1800) | 1500 |
| Echo Time (ms) | 40 | 33 ± 7 (30–40) |
| Pixel size (mm) | 1.98 ± 0.0 (1.96–2) | 1.81 ± 0.09 (1.72–1.88) |
| Section thickness (mm) | 5 | 5.6 ± 0.6 (5–6) |

Abbreviation: Data are expressed as mean ± standard deviation. Numbers in parenthesis are range. FLAIR = fluid-attenuated inversion recovery, DWI = diffusion weighted imaging, DSC = dynamic susceptibility contrast imaging

**2. Supplementary information: extraction of radiomic features**

Volume and shape features were used to describe the 3D geometric properties of the tumor, which included compactness, spherical disproportion, sphericity, surface area, and volume. The first-order features were derived from the intensity histogram using first-order statistics, including intensity range, energy, entropy, kurtosis, maximum, mean, median, uniformity, and variance. Textural features were obtained from a gray-level co-occurrence matrix (GLCM) and gray-level run-length matrix (GLRLM) ^1^ using 3D analyses of the tumor ROI in 13 directions of the 3D space. For GLCM analyses, texture features were computed for varying distances of 1, 2, 3 voxels in 13 directions. Wavelet transformation was applied with a single-level directional discrete wavelet transform of a high-pass and low-pass filter ^2^. In total, eight wavelet-decomposition images were generated from each MRI imaging sequence input: HHH, HHL, HLH, HLL, LHH, LHL, LLH, and LLL images, where ‘H’ was designated as a high-pass filter and ‘L’ is a low-pass filter. The first-order features and texture features were then applied to the wavelet-transformed images, (17 first-order features + 162 texture features) and multiplied by 8 images, to yield 1432 wavelet features.

The imaging features that were calculated were divided into three groups: 17 first-order statistics, 7 volume and shape-based features, and 162 texture features. Wavelet transformation was applied to the ADC and post-contrast T1-weighted images, and eight wavelet-decomposition images were generated from each MRI image that was input. Then first-order features and texture features were calculated from the eight wavelet decomposition images, which further resulted in 1432 wavelet features ([17 + 162] × 8). In total, 1618 features (17 first-order statistics, 7 volume and shape-based features, 162 texture features, and 1432 wavelet features) were obtained.

1. Volume and shape features

These features compute the three-dimensional size and shape of volume of interest $V(x,y,z)$. Let $Vol$ and $A$ denote the volume and surface area, respectively.

| **Surface area** | $A$ | **Compactness 2** | $36\pi\frac{A^{2}}{{Vol}^{3}}$ |
| --- | --- | --- | --- |
| **Volume** | $N*{pixel}_{x}*{pixel}_{y}*{pixel}_{z}$ | **Spherical disproportion** | $\frac{A}{\left( 6\sqrt{\pi}*Vol \right)^{\frac{2}{3}}}$ |
| **Surface to volume ratio** | $\frac{A}{Vol}$ | **Sphericity** | $\frac{\left( {6\pi}^{2}*Vol \right)^{\frac{2}{3}}}{A}$ |
| **Compactness 1** | $\frac{Vol}{\sqrt{\pi}*A^{\frac{2}{3}}}$ |  |  |

1. First-order statistics

First-order statistics were calculated from the histogram of voxel intensities, which represents the distribution of gray values within an image. Let P denote the first-order histogram of a volume of interest V(x,y,z) with isotropic voxel size and P*i* describe the number of voxels with gray level *i*. The number of gray-level bins set for *P* is represented as Ng. The *i*th probability vector of the first-order histogram is then defined as follows:

$$p\left( i \right)=\frac{P\left( i \right)}{\sum_{i=1}^{N_{g}} P\left( i \right)}$$

Let $\boldsymbol{V}_{ROI}$ and $\boldsymbol{V}$ denote the intensity values of all voxels within $V(x,y,z)$ with $N$ voxels and the whole image, respectively. The mean and center gray values within $V(x,y,z)$ are $\overline{V}$ and $\boldsymbol{C}$, respectively.

| **Number of voxels** | $\left\vert\boldsymbol{V}_{ROI} \right\vert$ | **Sum of intensities** | $\sum_{i}^{N} \boldsymbol{V}_{ROI}(i)$ |
| --- | --- | --- | --- |
| **Range** | $\text{max}\left( \boldsymbol{V}_{ROI} \right)-\text{min}\left( \boldsymbol{V}_{ROI} \right)$ | **Energy** | $N^{2}\sum_{i}^{N_{g}} {p\left( i \right)}^{2}$ |
| **Covered imageintensity range** | $\frac{\max\boldsymbol{V}_{ROI}-\min\boldsymbol{V}_{ROI}}{\left( \max\boldsymbol{V}-\min\boldsymbol{V}+eps \right)}$ | **Entropy** | $-\sum_{i}^{N_{g}} \left[ p\left( i \right)*\log_{2} \left( p\left( i \right)+eps \right) \right]$ |
| **Maximum intensity value** | $\max\left( \boldsymbol{V}_{ROI} \right)$ | **Kurtosis** | $\frac{\sum_{i}^{N_{g}} \left[ p\left( i \right)*\left( \boldsymbol{C}\left( i \right)- \overline{V} \right)^{4} \right]}{\left( \sum_{i}^{N_{g}} \left[ p\left( i \right)*\left( \boldsymbol{C}\left( i \right)-\overline{V} \right) \right]+eps \right)^{2}}$ |
| **Mean intensity value** | $\frac{1}{N}\sum_{i}^{N} \boldsymbol{V}_{ROI}(i)$ | **Skewness** | $\frac{\sum_{i}^{N_{g}} \left[ p\left( i \right)*\left( \boldsymbol{C}\left( i \right)- \overline{V} \right)^{3} \right]}{\left( \sum_{i}^{N_{g}} \left[ p\left( i \right)*\left( \boldsymbol{C}\left( i \right)- \overline{V} \right)^{3} \right]+eps \right)^{\frac{3}{2}}}$ |
| **Median intensity value** | $\mathrm{med} \left( \boldsymbol{V}_{ROI} \right)$ | **Root means square** | $\sum_{i}^{N_{g}} \left[ p\left( i \right)*\boldsymbol{C}\left( i \right)^{2} \right]$ |
| **Minimum intensity value** | $\min\left( \boldsymbol{V}_{ROI} \right)$ | **Variance** | $\frac{1}{N-1}\sum_{i}^{N} \left( \boldsymbol{V}_{ROI}\left( i \right)- \overline{V} \right)^{2}$ |
| **Mean absolute deviation** | $\sum_{i}^{N_{g}} \left[ p\left( i \right)*(\boldsymbol{C}\left( i \right)- \overline{V}) \right]$ | **Standard deviation** | $\sqrt{\frac{1}{N-1}\sum_{i}^{N} \left( \boldsymbol{V}_{ROI}\left( i \right)- \overline{V} \right)^{2}}$ |
| **Uniformity** | $\sum_{i}^{N_{g}} p\left( i \right)^{2}$ |  |  |

1. Texture features

Although first-order features provide information on the gray-level distribution of the volume of interest, they do not describe information related to the relative positions of the various gray levels of the volume of interest. The methods most often used for texture analysis are the gray level co-occurrence matrix (GLCM) and the gray level run length matrix (GLRLM). In the use of GLCM, various textural features are extracted, and GLRLM characterizes coarse textures as having many pixels in a constant gray level run and fine textures as having few pixels in such a run. Both GLCM and GLRLM are matrix-based features as well as being constructed from 3D analysis of a volume of interest with 26-voxel connectivity, which are considered neighbors in all 13 directions in 3D.

- 1. GLCM features

Let $G$ denote the GLCM of a quantized volume $V(x,y,z)$ with isotropic voxel size and let $G_{\alpha,\delta}(i,j)$ represent the number of times that voxels of gray level i were neighbors with voxels of gray level $j$ in $V(x,y,z)$ in one of 13 directions of $\alpha$ and at a distance $\delta=1,2,3$. GLCM is the size of $N_{g}\times N_{g}$ where $N_{g}$ describes a pre-defined number of quantized gray level sets in $V(x,y,z)$. For each direction $\alpha$ and distance $\delta$, the normalized GLCM is obtained as follows:

$g_{\alpha,\delta}\left( i,j \right)=g\left( i,j \right)=\frac{G\left( i,j \right)}{\sum_{i=1}^{N_{g}} \sum_{j=1}^{N_{g}} G\left( i,j \right)}$.

Frequently used feature quantities for each direction and distance are also defined as follows:

- $\mu$ is the mean of $g\left( i,j \right)$
- $\sigma$ is the standard deviation of $g\left( i,j \right)$
- $g_{x}\left( i \right)$ is the marginal row probability of $g\left( i,j \right)$: $g_{x}\left( i \right)=\sum_{j}^{N_{g}} g\left( i,j \right)$,
- $\mu_{x}$ is the mean of $g_{x}\left( i \right)$
- $\sigma_{x}$ is the standard deviation of $g_{x}\left( i \right)$

| Mean and standard deviation of the followings for 13 directions and 3 distances | | | |
| --- | --- | --- | --- |
| **autocorrelation** | $\sum_{i}^{N_{g}} \sum_{j}^{N_{g}} i*j*g(i,j)$ | **Haralick correlation** | $\frac{1}{\sigma_{x}}\sum_{i}^{N_{g}} \sum_{j}^{N_{g}} \left( i*j*g\left( i,j \right) \right)-\mu_{x}$ |
| **cluster prominence** | $\sum_{i}^{N_{g}} \sum_{j}^{N_{g}} \left( i+j-2\mu\right)^{4}*g\left( i,j \right)$ | **inverse difference** | $\sum_{i}^{N_{g}} \sum_{j}^{N_{g}} \frac{g\left( i,j \right)}{1+\left\vert i-j \right\vert}$ |
| **cluster shade** | $\sum_{i}^{N_{g}} \sum_{j}^{N_{g}} \left( i+j-2\mu\right)^{3}*g\left( i,j \right)$ | **inverse difference normalized** | $\frac{1}{N_{g}}\sum_{i}^{N_{g}} \sum_{j}^{N_{g}} \frac{g\left( i,j \right)}{1+\left\vert i-j \right\vert}$ |
| **cluster tendency** | $\sum_{i}^{N_{g}} \sum_{j}^{N_{g}} \left( i+j-2\mu\right)^{2}*g\left( i,j \right)$ | **inverse difference moment** | $\sum_{i}^{N_{g}} \sum_{j}^{N_{g}} \frac{g\left( i,j \right)}{1+\left( i-j \right)^{2}}$ |
| **contrast** | $\sum_{i}^{N_{g}} \sum_{j}^{N_{g}} \left( i-j \right)^{2}*g\left( i,j \right)$ | **inverse difference moment normalized** | $\frac{1}{{N_{g}}^{2}}\sum_{i}^{N_{g}} \sum_{j}^{N_{g}} \frac{g\left( i,j \right)}{1+\left( i-j \right)^{2}}$ |
| **correlation** | $\frac{1}{\sigma}\sum_{i}^{N_{g}} \sum_{j}^{N_{g}} \left( i-\mu\right)\left( j-\mu\right)*g\left( i,j \right)$ | **inverse variance** | $\sum_{i}^{N_{g}} \sum_{j}^{N_{g}} \frac{g\left( i,j \right)}{\left( i-j \right)^{2}}$ |
| **difference average** | $\sum_{k}^{N_{g}} k*g_{x-y}\left( k \right)$ | **maximum probability** | $max\left( g\left( i,j \right) \right)$ |
| **difference entropy** | $-\sum_{k}^{N_{g}} g_{x-y}\left( k \right)*{log}_{2}\left( g_{x-y}\left( k \right)+eps \right)$ | **sum average** | $\sum_{k}^{2N_{g}} i*g_{x+y}\left( k \right)$ |
| **difference variance** | $\sum_{k}^{N_{g}} \left( k-\bar{g_{x-y}} \right)^{2}*g_{x-y}\left( k \right)$ | **sum entropy** | $-\sum_{k}^{2N_{g}} g_{x+y}\left( k \right)*{log}_{2}\left( g_{x+y}\left( k \right)+eps \right)$ |
| **dissimilarity** | $\sum_{i}^{N_{g}} \sum_{j}^{N_{g}} \left\vert i-j \right\vert*g\left( i,j \right)$ | **sum variance** | $\sum_{k}^{2N_{g}} \left( k-\bar{g_{x+y}} \right)^{2}*g_{x+y}\left( k \right)$ |
| **energy** | $\sum_{i}^{N_{g}} \sum_{j}^{N_{g}} {g\left( i,j \right)}^{2}$ | **variance** | $\sum_{i}^{N_{g}} \sum_{j}^{N_{g}} \left( i-\mu\right)^{2}g\left( i,j \right)$ |
| **entropy** | $-\sum_{i}^{N_{g}} \sum_{j}^{N_{g}} g\left( i,j \right)*{log}_{2}\left( g\left( i,j \right)+eps \right)$ |  |  |

- 1. GLRLM features

Let $Q$ denote the GLRLM of a quantized volume $V(x,y,z)$ with isotropic voxel size and $Q(i,j)$ represent the number of runs of gray level *i* with $j$ consecutive voxels in 1 of the 13 directions of $\alpha$. GLRLM is the size of $N_{g}\times N_{l}$ where $N_{g}$ describes the pre-defined number and $N_{l}$ represents the length of the longest run of quantized gray level sets in $V(x,y,z)$. $N_{p}$ is the number of voxels in $V(x,y,z)$.

| Mean and standard deviation of the followings for 13 directions and 3 distances | | | |
| --- | --- | --- | --- |
| **number of runs** | $N_{run}$ | **low gray level run emphasis** | $\frac{1}{N_{run}}\sum_{i}^{N_{g}} \sum_{j}^{N_{l}} \frac{1}{i^{2}}*Q\left( i,j \right)$ |
| **gray level nonuniformity** | $\frac{1}{N_{run}}\sum_{i}^{N_{g}} \left( \sum_{j}^{N_{l}} Q\left( i,j \right) \right)^{2}$ | **run length nonuniformity** | $\frac{1}{N_{run}}\sum_{j}^{N_{l}} \left( \sum_{i}^{N_{g}} Q\left( i,j \right) \right)^{2}$ |
| **high gray level run emphasis** | $\frac{1}{N_{run}}\sum_{i}^{N_{g}} \sum_{j}^{N_{l}} i^{2}*Q\left( i,j \right)$ | **run percentage** | $\frac{N_{run}}{N_{p}}$ |
| **long run emphasis** | $\frac{1}{N_{run}}\sum_{i}^{N_{g}} \sum_{j}^{N_{l}} j^{2}*Q\left( i,j \right)$ | **short run emphasis** | $\frac{1}{N_{run}}\sum_{i}^{N_{g}} \sum_{j}^{N_{l}} \frac{1}{j^{2}}*Q\left( i,j \right)$ |
| **long run high gray level emphasis** | $\frac{1}{N_{run}}\sum_{i}^{N_{g}} \sum_{j}^{N_{l}} {i^{2}*j}^{2}*Q\left( i,j \right)$ | **short run high gray level emphasis** | $\frac{1}{N_{run}}\sum_{i}^{N_{g}} \sum_{j}^{N_{l}} \frac{i^{2}}{j^{2}}*Q\left( i,j \right)$ |
| **long run low gray level emphasis** | $\frac{1}{N_{run}}\sum_{i}^{N_{g}} \sum_{j}^{N_{l}} \frac{j^{2}}{i^{2}}*Q\left( i,j \right)$ | **short run low gray level emphasis** | $\frac{1}{N_{run}}\sum_{i}^{N_{g}} \sum_{j}^{N_{l}} \frac{1}{i^{2}*j^{2}}*Q\left( i,j \right)$ |

**Supplementary Table 2.** Selection of clinical predictors from the training set using a univariate Cox hazard regression model.

| **Predictors** | **Hazard ratio** | **95% Confidence interval** | ***P*-value** |
| --- | --- | --- | --- |
| Age | 1.02 | 1.01–1.04 | **0.039** |
| Sex | 0.88 | 0.61–1.30 | 0.543 |
| KPS at treatment initiation | 1.70 | 1.02–2.86 | **0.043** |
| Location | 0.77 | 0.52–1.12 | 0.167 |
| Volume | 1.00 | 0.99–1.00 | 0.767 |
| Gross total resection vs. biopsy and partial resection | 0.67 | 0.53–0.84 | **0.0004** |
|  |  |  |  |

Abbreviation: KPS, Karnofsky performance score; CCRT, concurrent chemoradiation therapy; TMZ, temozolomide. KPS score was binary with 1) score same or above 70 and 2) below 70.

**Supplementary Figure 1.** Multiparametric magnetic resonance radiomics feature selection using the least absolute shrinkage and selection operator (LASSO) Cox regression model.

(A) Identification of the optimal regularization parameter (λ) in the LASSO model used 10-fold cross-validation. As a result, a λ value between the minimum criterion of 0.159 and one standard error of 0.292 was selected. The dotted vertical line was plotted at the value selected using 10-fold cross-validation, for which the optimal λ resulted in 6 significant non-zero coefficients.

(B) LASSO coefficient profiles using the 6472 derived multiparametric radiomics features.


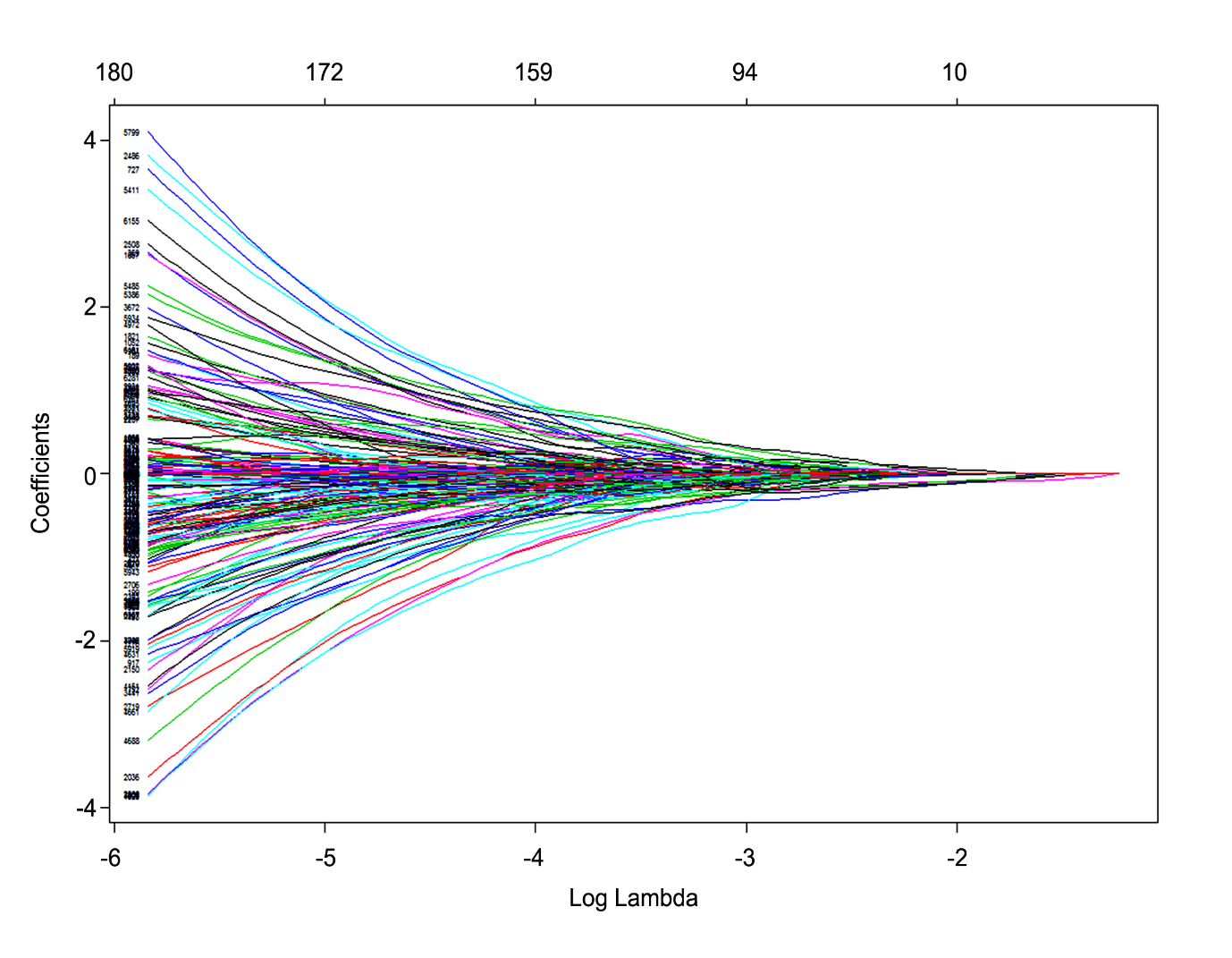

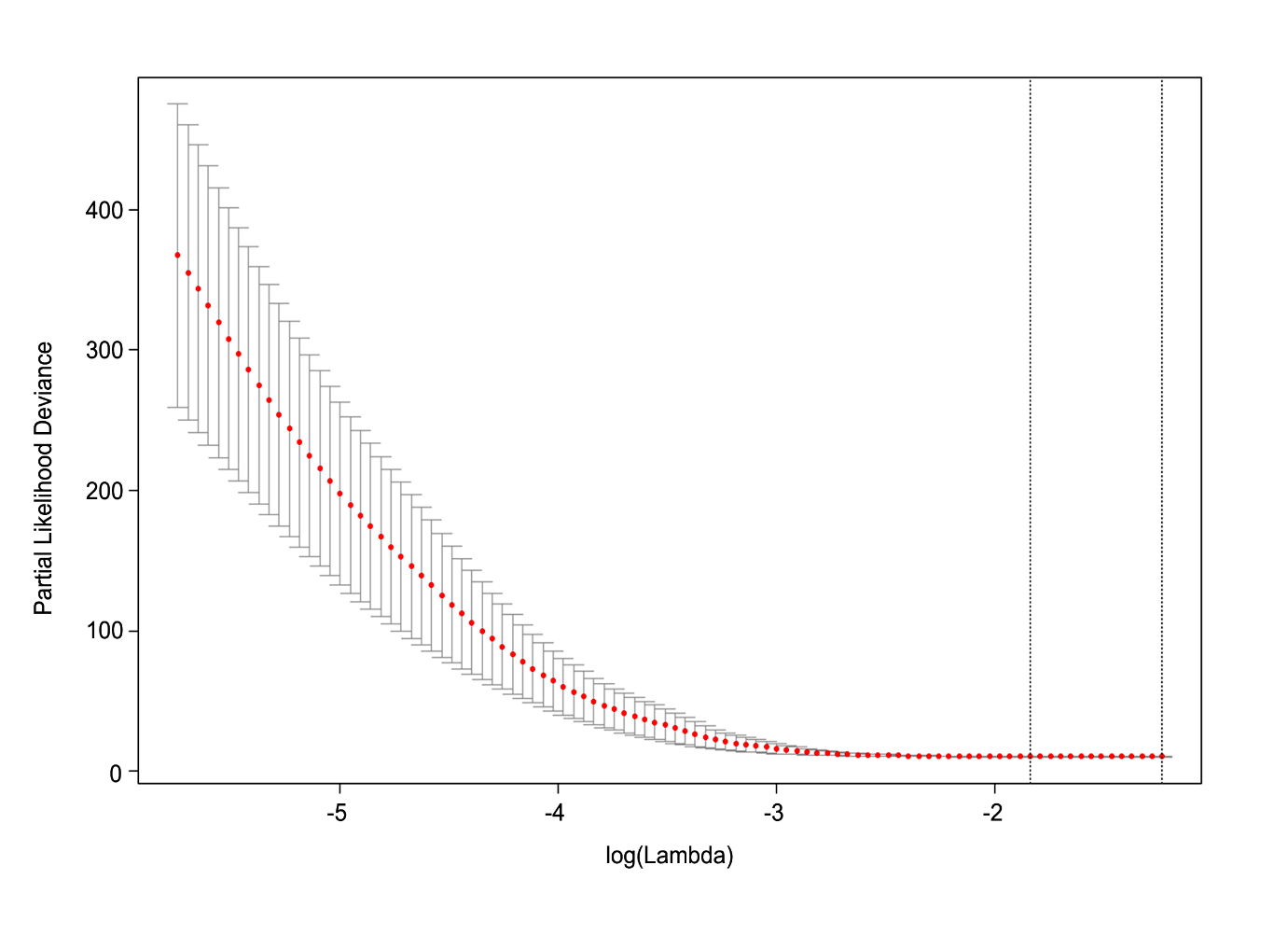


**References**

1. Materka, A. & Strzelecki, M. Texture Analysis Methods–A Review.

2. Wang, J. Z. Wavelets and imaging informatics: A review of the literature. *Journal of Biomedical Informatics* **34**, 129-141 (2001).
